# A meta-analysis of the correlation between the duration of the second stage of labor and recent postpartum pelvic floor injury in primiparous

**DOI:** 10.3389/fmed.2025.1567774

**Published:** 2025-07-31

**Authors:** Yuying Chen, Bin Zhao, Zhaoyuan Chen

**Affiliations:** ^1^Department of Gynecology and Obstetrics, Binzhou People’s Hospital, Binzhou, China; ^2^Department of Orthopedics, Binzhou People’s Hospital, Binzhou, China

**Keywords:** recent pelvic floor dysfunction, duration of the second stage of labor, primiparous women, meta-analysis, correlation

## Abstract

**Objective:**

This study aimed to explore the correlation between the duration of the second stage of labor and recent postpartum pelvic floor injury in primiparous women.

**Methods:**

Randomized controlled trials (RCTs) were systematically evaluated by a comprehensive literature review and inclusion criteria. This study aimed to evaluate the correlation between the duration of the second stage of labor and recent postpartum pelvic floor dysfunction in primiparous women. The quality of the included studies was assessed using the Cochrane risk of bias tool, and meta-analysis was performed using RevMan 5.3 software. Primary outcome measures included pelvic organ prolapse (POP), urinary incontinence (UI), fecal incontinence (FI), postpartum sexual dysfunction, pelvic floor pain, and urinary retention.

**Results:**

A total of 13 articles were included, comprising 2,862 researchers: 1,407 in the extension group, and 1,455 in the general group. All studies were randomized controlled trials. There was no significant difference in POP between the extension group and the general group [OR = 1.25, 95% CI (0.99–1.59), *p* = 0.06]. UI was more severe in the extension group [OR = 1.95, 95% CI (1.30–2.92), *p* = 0.001]. There was no significant difference in FI between the extension and control groups [OR = 1.28, 95% CI (0.98–1.68), *p* = 0.07]. Postpartum sexual dysfunction was not significantly worse in the extension group than in the control group [OR = 1.67, 95% CI (0.81–3.42), *p* = 0.16]. Pelvic floor pain was stronger in the extension group [OR = 1.24, 95% CI (1.03–1.50), *p* = 0.03]. Urinary retention was more severe in the extension group [OR = 2.40, 95% CI (1.12–5.15), *p* = 0.03].

**Conclusion:**

The prolongation of the second stage of labor in primiparous parturients is significantly correlated with recent postpartum pelvic floor dysfunction, including urinary incontinence, pelvic floor pain, and urinary retention. Although no significant differences were observed in pelvic organ prolapse and fecal incontinence, a trend was observed. Postpartum sexual dysfunction did not show a significant correlation with prolonged labor.

## 1 Introduction

In the field of obstetrics and gynecology, the delivery process of primiparous women has always been a focus of research, with particular attention given to the second stage of labor. The second stage of labor spans from complete dilation of the orifice of the uterus to the delivery of the fetus and presents significant physical and physiological challenges for primiparous women (1). With the advancement of modern medical care and increased attention given to maternal health, it is gradually recognized that the delivery process profoundly impacts maternal pelvic floor function. In particular, recent postpartum pelvic floor function injuries are not only related to maternal health but also affect women’s quality of life. The importance of pelvic floor function for women cannot be ignored. It supports the bladder, uterus, rectum, and other vital organs in the pelvis, maintaining their normal position and function (2). Normal pelvic floor function is essential for the physiological activities of urination, defecation, and sexual function in women. However, during childbirth, particularly in the second stage of labor, the pelvic floor tissues may be damaged to different degrees, resulting in a series of pelvic floor dysfunction problems. Recent postpartum pelvic floor dysfunction is a complex and diverse problem, primarily manifested as pelvic organ prolapse (POP), urinary incontinence (UI), fecal incontinence (FI), postpartum sexual dysfunction, pelvic floor pain, and urinary retention (3). Pelvic organ prolapse may cause parturients to feel lower abdominal bulge and vaginal mass prolapse, which can seriously affect their daily life and activities (4). Urinary incontinence can lead to involuntary urine leakage in parturient women, particularly during activities that increase abdominal pressure, such as coughing, laughing, and sneezing (5). Fecal incontinence not only causes physical discomfort to parturients but may also cause perianal skin problems (6). After the pelvic floor muscles, fascia, and ligaments are damaged during delivery, they may become either loose or tense, leading to pain during sexual intercourse (7). Pelvic floor pain causes parturients to experience pain while sitting, walking, or having sex, thus reducing the quality of life (8). Uroschesis can result in excessive bladder filling, which may lead to a series of urinary system problems (9). The impact of primiparous women’s experience during the second stage of labor on pelvic floor function is multifactorial. Moreover, the duration of labor is one of the key factors. A prolonged second stage of labor indicates that the pelvic floor muscles, fascia, and ligaments are compressed and stretched by the fetus for an extended period, which may exceed their physiological tolerance, leading to tissue damage and structural changes. For example, when the fetal head stays on the pelvic floor for an extended period of time, continuous pressure is exerted on the pelvic floor tissue, similar to an excessively stretched rubber band. This may compromise the elasticity of the tissues, preventing them from returning to their original state. Such injuries may manifest as obvious pelvic floor dysfunction in the near future after childbirth, causing physical and mental pain for primiparous women.

At present, many studies have explored the correlation between the duration of the second stage of labor of primiparous women and recent postpartum pelvic floor function impairment, but the results have some differences. Differences in sample selection, research methods, and evaluation indicators among studies make it difficult for clinicians and researchers to accurately understand the relationship between the two. Therefore, a comprehensive analysis of existing randomized controlled trials (RCTs) through comprehensive literature review, systematic evaluation, and meta-analysis is of great significance. It helps identify the correlation between the duration of the second stage of labor and recent postpartum pelvic floor function impairment in primiparous women. This, in turn, provides a more reliable basis for clinical practice, enhances prevention and treatment strategies for postpartum pelvic floor function impairment, and supports the health and quality of life of primiparous women.

## 2 Data and methods

### 2.1 Inclusion and exclusion criteria

#### 2.1.1 Inclusion criteria

The inclusion criteria were as follows: (1) study type: RCTs only; (2) Research population: primiparous women of a defined age range, with no pregnancy complications (such as, severe hypertensive disorders of pregnancy or gestational diabetes mellitus with severe microangiopathy) and normal pregnancies; (3) intervention approach: accurate recording of the duration of the second stage of labor; and (4) outcome measures: inclusion of at least one of the following—POP, UI, FI, pelvic pain, or urinary retention. Outcomes were evaluated according to scientific and standardized methods within 6 weeks after delivery.

#### 2.1.2 Exclusion criteria

The exclusion criteria were as follows: (1) study types other than non-randomized controlled trials; (2) parturients with severe pregnancy complications affecting pelvic floor function or primiparous women with multiple pregnancies; (3) studies lacking accurate data on the duration of the second stage of labor or using unknown assessment methods for assessing pelvic floor function impairment; and (4) studies with significant confounding factors that were not effectively controlled or adjusted.

### 2.2 Research method

#### 2.2.1 Retrieval strategy

Literature searches were conducted simultaneously in the PubMed (including MEDLINE), Web of Science, Cochrane Library, and EMBASE databases. A unified search strategy was employed using the following terms: (“Second stage of labor”) AND (“Pelvic floor dysfunction” OR “Pelvic organ prolapse” OR “Urinary incontinence” OR “Fecal incontinence” OR “Postpartum sexual dysfunction” OR “Pelvic Floor Pain” OR “urinary retention”), with the search duration limited to studies from database inception up to October 2024 and restricted to randomized controlled trials (RCTs). PubMed matched MeSH terms [such as “Second stage of labor”(Mesh)] through advanced search and enabled “Clinical Queries” to accurately filter RCTs; Web of Science searched the core collection “Topic” field and then used “Research Area” to filter literature related to obstetrics and gynecology; Cochrane Library combined MeSH with free text, optimizing the search using systematic review professional syntax; EMBASE mapped standard terms through the EMTREE thesaurus and performed full-field free text expansion. Searches in each database included title/abstract/keywords fields, excluding non-research literature such as conference abstracts and reviews. Finally, duplicates were removed using EndNote X9 before entering the screening process.

#### 2.2.2 Literature screen and data extraction

Literature screening was conducted independently by at least two investigators. Significantly unrelated articles were first excluded based on the title and abstract. Full texts were then obtained for further evaluation of potentially relevant studies. Data extracted included basic information about the study (author, year of publication, etc.), sample size, and duration of labor. In cases where consensus could not be reached, a third investigator participated in the decision-making process.

#### 2.2.3 Literature screen and quality evaluation

**Figure 1:**
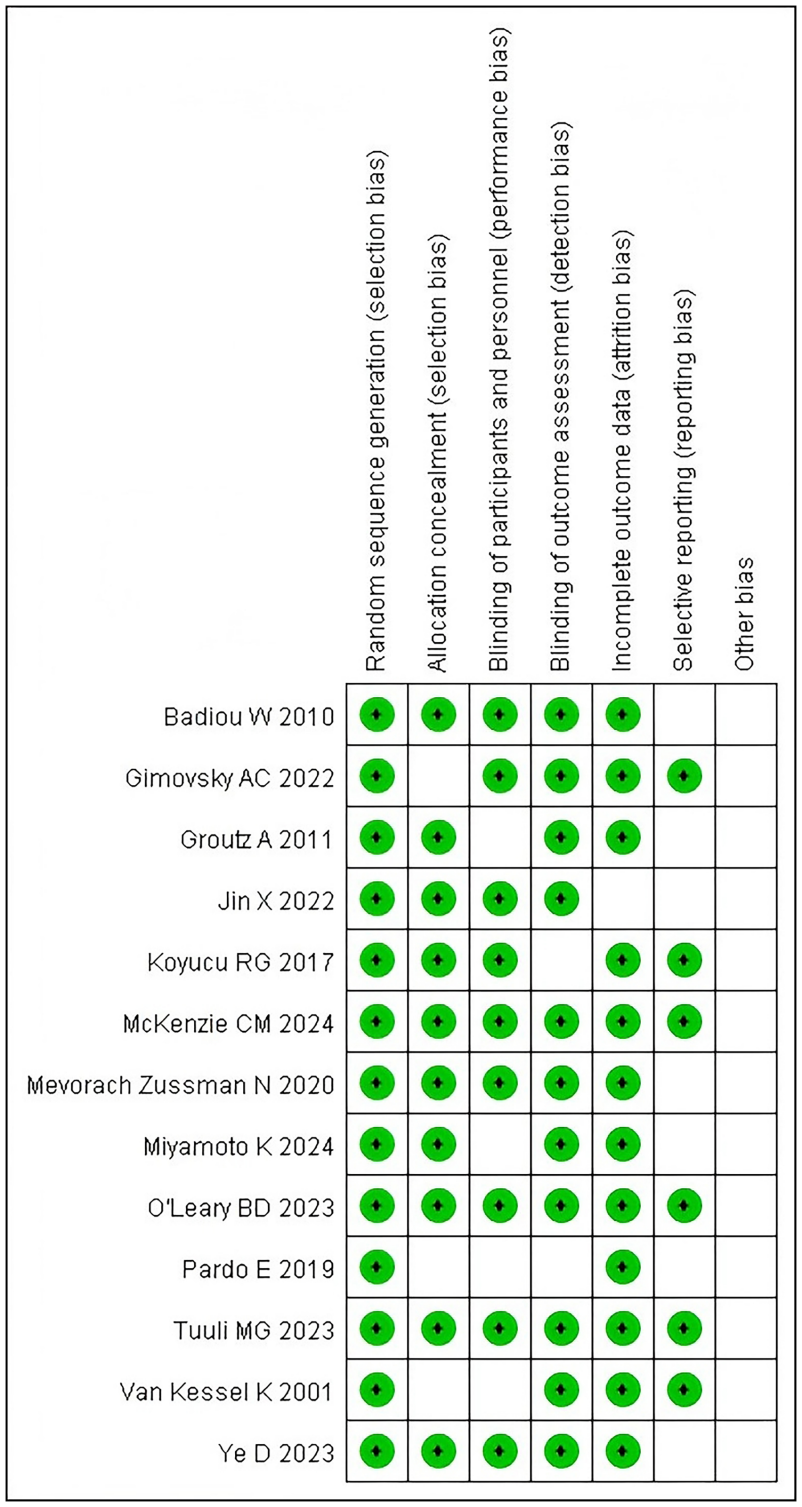
Summary chart of bias risk assessment of 13 literatures.

Using the Cochrane risk of bias tool, two independent reviewers assessed the study quality. For example, Wright et al. (10) demonstrated low selection bias through randomized allocation but had high detection bias due to unblinded outcome assessment. MacCraith et al. (11) exhibited high attrition bias (25% dropout rate) without intention-to-treat analysis. Full assessments are presented in Figure 1. The aspects of evaluation included random sequence generation, allocation concealment, blinded implementation, integrity of outcome data, selective reporting of study results, and other potential sources of bias. Conclusions from the quality evaluation, such as low-risk, high-risk, or unclear, were presented for each included article.

#### 2.2.4 PROSPERO registration and PRISMA declaration

This systematic review was prospectively registered in the PROSPERO International Prospective Register of Systematic Reviews (Registration number: CRD420251056890). The study protocol follows the Preferred Reporting Items for Systematic Reviews and Meta-Analyses (PRISMA) 2020 statement.

### 2.3 Statistical analysis

Heterogeneity was assessed using Cochran’s *Q*-test (significance threshold: *p* < 0.10) and quantified with the *I*^2^ statistic. We defined substantial heterogeneity as *I*^2^ > 50%, with a 95% confidence interval (CI). For outcomes showing substantial heterogeneity (*I*^2^ > 50%), sensitivity analyses were performed using a random-effects model and subgroup analyses were stratified by delivery mode (spontaneous vs. assisted) and epidural analgesia use. We planned to conduct subgroup analyses based on the definition of “prolonged second stage of labor” (e.g., >2 h vs. >3 h). However, these analyses were not feasible due to insufficient data, as most studies used a cutoff of >2 h and did not report data separately for other durations. For urinary incontinence, significant heterogeneity was observed (*I*^2^ = 60%), indicating substantial heterogeneity. Therefore, a random-effects model was used for the meta-analysis of this outcome. For pelvic organ prolapse, where heterogeneity was not significant (*I*^2^ = 20%), a fixed-effect model was used. Publication bias was evaluated using Egger’s regression test for outcomes with ≥10 studies; for outcomes with <10 studies, we acknowledge the limitation of insufficient power in detecting bias. All analyses were conducted using RevMan 5.3 and R (Metafor package).

## 3 Results

### 3.1 Literature retrieval results

A total of 6,417 English articles were retrieved from relevant databases. After removing duplicates, 5,120 articles remained. Subsequently, 3,100 non-controlled studies and 1,895 articles that were not prospectively randomized controlled trials, had unclear patient data or observation times, or involved non-primiparous women were excluded. According to the inclusion and exclusion criteria, 13 articles were finally included. The literature retrieval process is shown in Figure 1.

### 3.2 Basic characteristics of the included articles

A total of 13 articles were included, involving 2,862 researchers—1,407 cases in the extension group and 1,455 cases in the control group. All studies were randomized controlled experiments. The basic characteristics of the included articles are shown in Table 1.

**Table 1:** Basic characteristics of the included articles.

Article (year)	Sample size		Second labor duration		Evaluating indicator	Grouping method
	Extension group	Normal group	Extension group	Normal group		
McKenzie et al. (17)	384	383	>60 min	≤60 min	POP, FI, PFP	Random control
Tuuli et al. (18)	489	452	>60 min	≤60 min	POP, FI, PFP	Random control
Gimovsky et al. (19)	17	17	>60 min	≤60 min	POP, UI, PSD, PFP	Random control
Mevorach Zussman et al. (20)	27	54	134.1 ± 74.6^a^	73.4 ± 71.6^a^	POP, UI, FI, PSD, PFP	Random control
Van Kessel et al. (21)	85	88	> 60 min ≤	60 min	UI	Random control
Koyucu et al. (22)	40	40	63.2 ± 21.3^a^	46.6 ± 23.4^a^	POP	Random control
Badiou et al. (23)	98	99	>120 min	60–120 min	UI, PSD	Random control
O’Leary et al. (24)	99	98	60–120 min	≤60 min	UI, PSD	Random control
Jin et al. (25)	31	30	>120 min	≤120 min	UI	Random control
Pardo et al. (26)	46	46	>120 min	≤120 min	PFP	Random control
Groutz et al. (27)	41	82	>120 min	≤120 min	UI, UR	Random control
Miyamoto et al. (28)	49	52	>60 min	≤60 min	UR	Random control
Ye et al. (29)	12	16	81 ± 46.5^a^	46 ± 25.0^a^	UR	Random control

PSD, postpartum sexual dysfunction; PFP, pelvic floor pain; UR, urinary retention.

a Indicates that the difference of measurement data is significant.

### 3.3 Quality evaluation of the included literature

**Figure 2:**
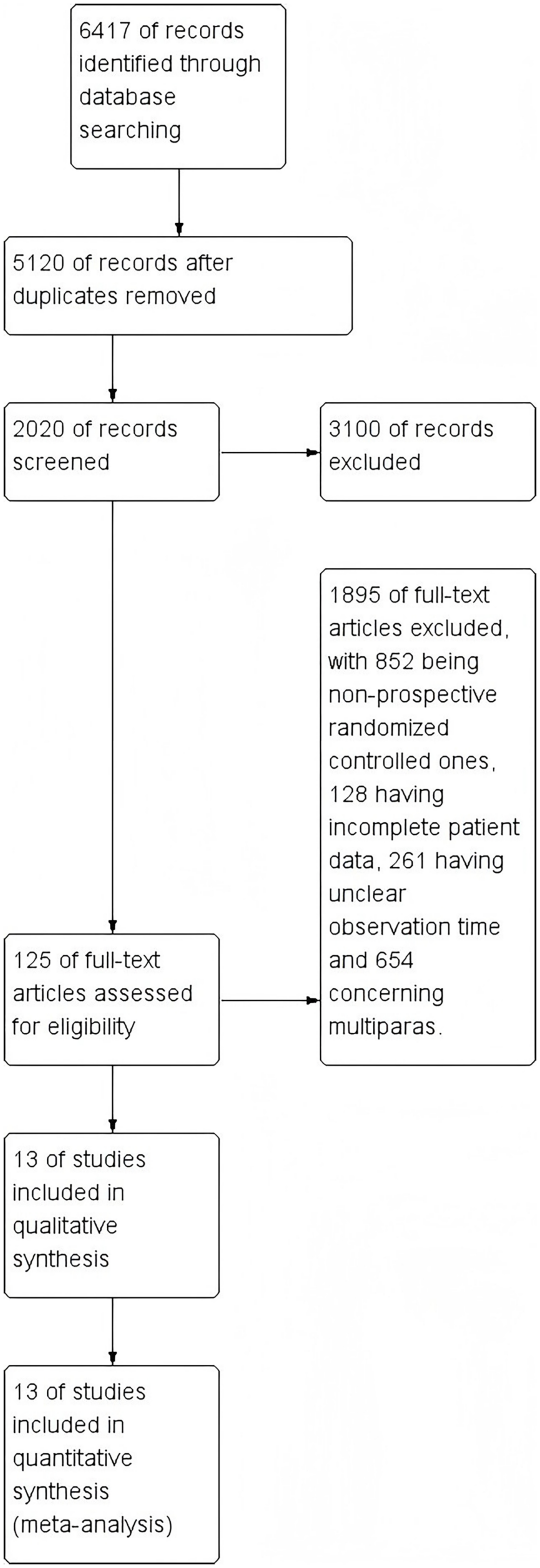
Document retrieval flowchart.

All 13 studies were classified as having an “unknown risk” because no other apparent risk source was found in any of the articles (see Figure 2).

### 3.4 META-analysis results

#### 3.4.1 POP

**Figure 3:**
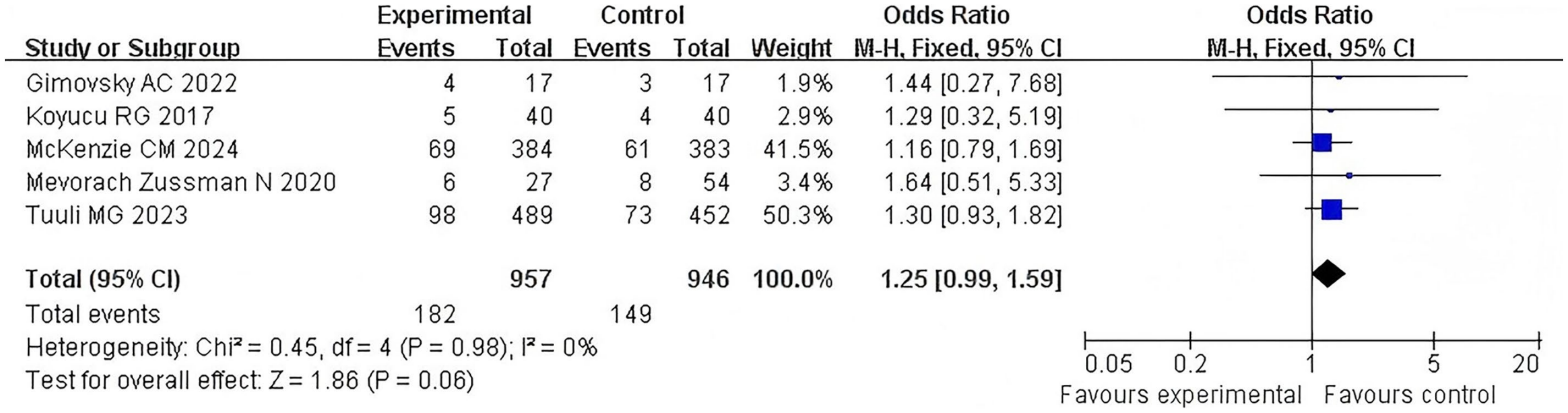
POP comparison forest map.

Five articles examined the correlation between the duration of the second stage of labor for primiparous women and the recent postpartum pelvic organ prolapse, and the POP-Q score of stage II or above is taken as an effective index. The *Q*-test and *I*^2^-test revealed *p* = 0.98 (chi-squared = 0.45, df = 4) and *I*^2^ = 0%, indicating that there was no clear heterogeneity between the studies. Data analysis was conducted using the Peto fixed-effect method, and the odds ratio (OR) was calculated. As a result, the difference between the two groups was not significant (*Z* = 1.86, *p* = 0.06). POP was not more severe in the extension group compared to the control group, OR = 1.25, 95% CI (0.99–1.59), and *p* = 0.06. Figure 3 shows the forest plot illustrating the association between prolonged second stage of labor and pelvic organ prolapse (OR = odds ratio and CI = confidence interval). An OR greater than 1 indicates an increased risk of pelvic organ prolapse in women with a prolonged second stage of labor. The diamond in the plot represents the pooled odds ratio (see Figure 3).

#### 3.4.2 UI

**Figure 4:**
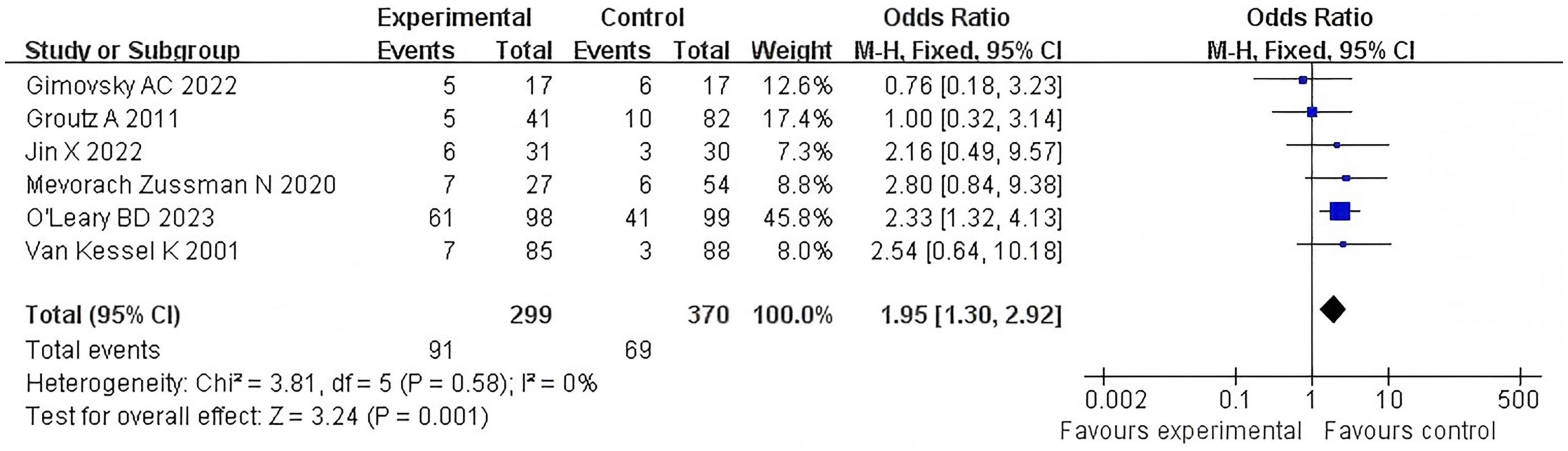
UI comparison forest map.

Six articles examined the correlation between the duration of the second stage of labor of primiparous women and recent postpartum urinary incontinence, with a UDI-6 score of more than 8 points as an effective indicator. The *Q*-test and *I*^2^-test revealed *p* = 0.58 (chi-squared = 3.81, df = 5) and *I*^2^ = 0%, indicating that there was no clear heterogeneity between the studies. The results showed that the difference between the two groups was statistically significant (*Z* = 3.24, *p* = 0.001). Moreover, the UI condition was more severe in the extension group than the control group [OR = 1.95, 95% CI (1.30–2.92), *p* = 0.001]. Figure 4 shows the forest map of the association between prolonged second stage of labor and urinary incontinence. OR greater than 1 indicates that women with prolonged second stage of labor have an increased risk of urinary incontinence. The diamond in the forest map represents the combined odds ratio (see Figure 4).

#### 3.4.3 FI

**Figure 5:**
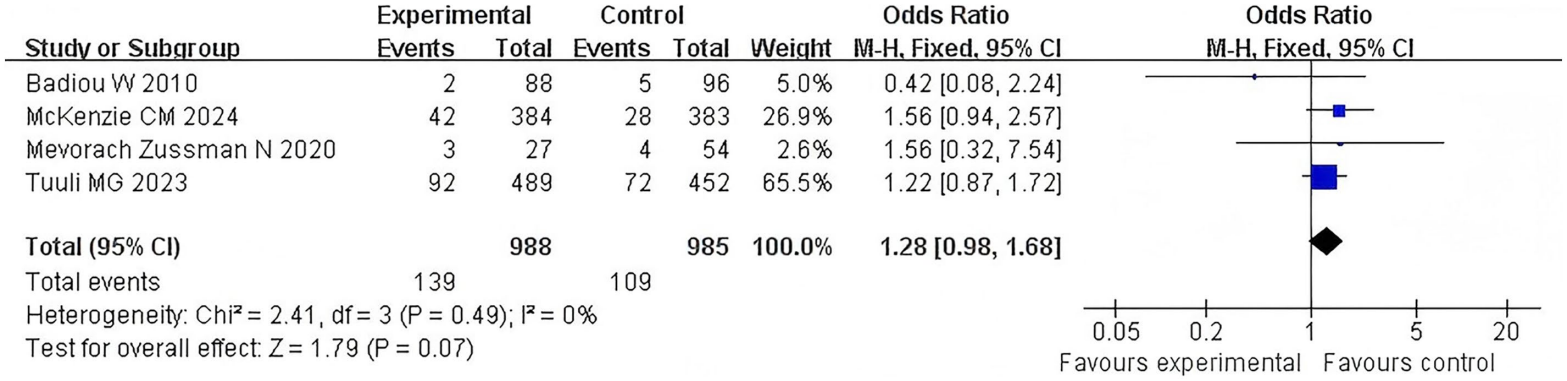
FI comparison forest map.

Four articles examined the correlation between the duration of the second stage of labor of primiparous women and recent postpartum fecal incontinence, and an increase of more than 4 points in the Fecal Incontinence Severity (FISI) score was used as an effective index. The *Q*-test and *I*^2^-test showed *p* = 0.49 (chi-squared = 2.41, df = 3) and *I*^2^ = 0%, indicating that there was no heterogeneity between the studies. The results showed that there was no significant difference between the two groups (*Z* = 1.79, *p* = 0.07). Furthermore, the FI condition in the extension group was not significantly worse than that in the control group [OR = 1.28, 95% CI (0.98–1.68), *p* = 0.07]. In Figure 5, the forest plot shows the association between prolonged second stage of labor and fecal incontinence. An OR greater than 1 indicates an increased risk of fecal incontinence in women with a prolonged second stage of labor. The diamond in the plot represents the pooled odds ratio. See Figure 5 for details.

#### 3.4.4 Postpartum sexual dysfunction

**Figure 6:**
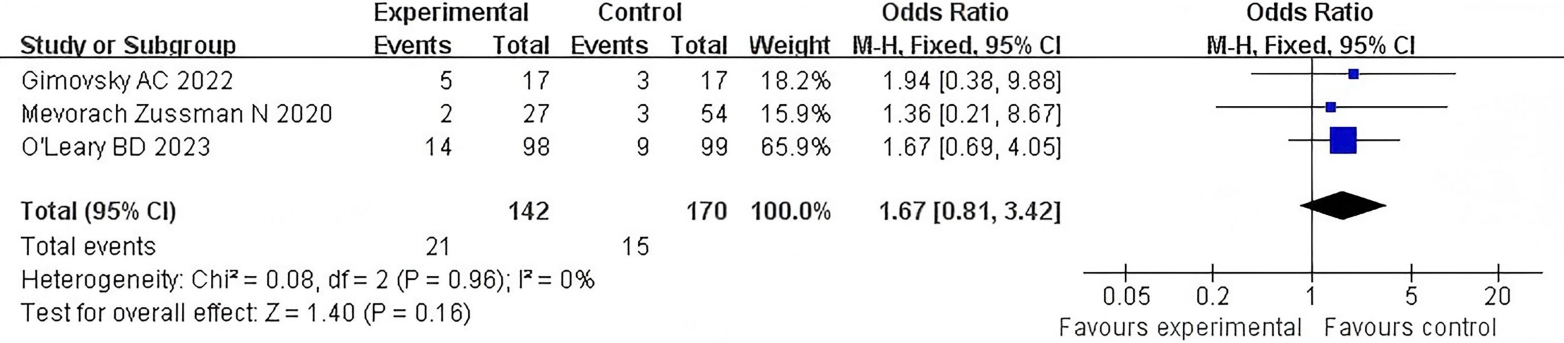
Postpartum sexual dysfunction comparison forest map.

Three articles examined the correlation between the duration of the second stage of labor of primiparous women and recent postpartum sexual dysfunction. The *Q*-test and *I*^2^-test showed *p* = 0.96 (chi-squared = 0.08, df = 2), and *I*^2^ = 0%, indicating that there was no heterogeneity between the studies. The results showed that there was no significant difference between the two groups (*Z* = 1.40, *p* = 0.16). In addition, sexual dysfunction in the extension group was not significantly worse than that in the control group [OR = 1.67, 95% CI (0.81–3.42), *p* = 0.16]. In Figure 6, the forest plot shows the association between prolonged second stage of labor and postpartum sexual dysfunction. An OR greater than 1 indicates an increased risk of postpartum sexual dysfunction in women with a prolonged second stage of labor. The diamond in the plot represents the pooled odds ratio. See Figure 6 for details.

#### 3.4.5 Pelvic floor pain

**Figure 7:**
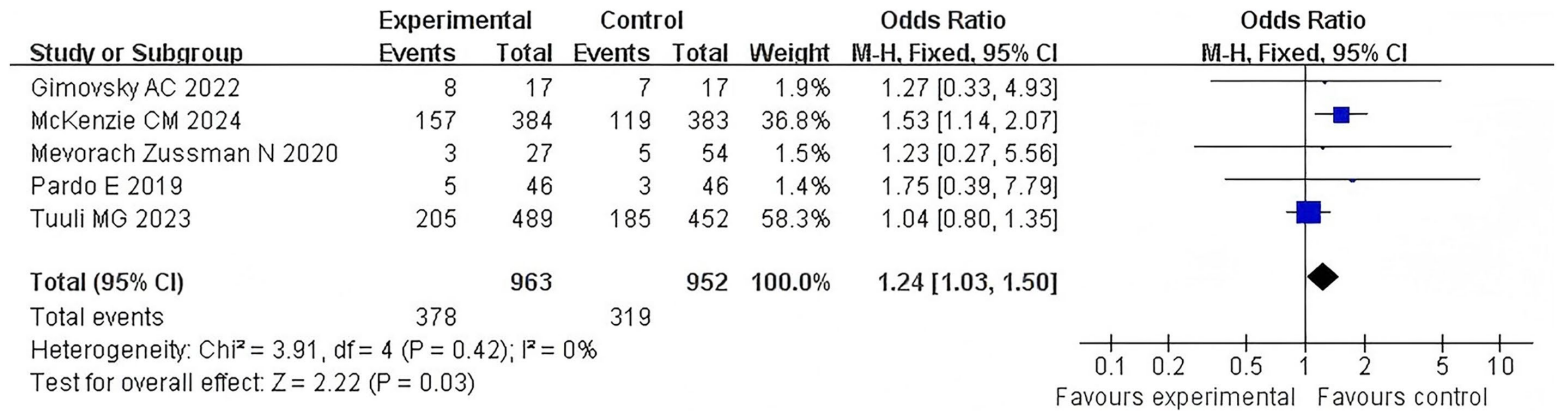
Pelvic floor pain comparison forest map.

Five articles examined the correlation between the duration of the second stage of labor in primiparous women and recent postpartum pelvic floor pain, using an increase of more than 4 points in the PFDI-20 score as an effective indicator. The *Q*-test and *I*^2^-test showed *p* = 0.42 (chi-squared = 3.91, df = 4) and *I*^2^ = 0%, indicating no heterogeneity among the studies. The results showed no significant difference between the two groups (*Z* = 2.22, *p* = 0.03). Moreover, pelvic floor pain in the extension group was significantly more severe than that in the control group [OR = 1.24, 95% CI (1.03–1.50), *p* = 0.03]. In Figure 7, the forest plot shows the association between prolonged second stage of labor and pelvic floor pain. An OR greater than 1 indicates an increased risk of pelvic floor pain in women with a prolonged second stage of labor. The diamond in the plot represents the pooled odds ratio. See Figure 7 for details.

#### 3.4.6 Urinary retention

**Figure 8:**
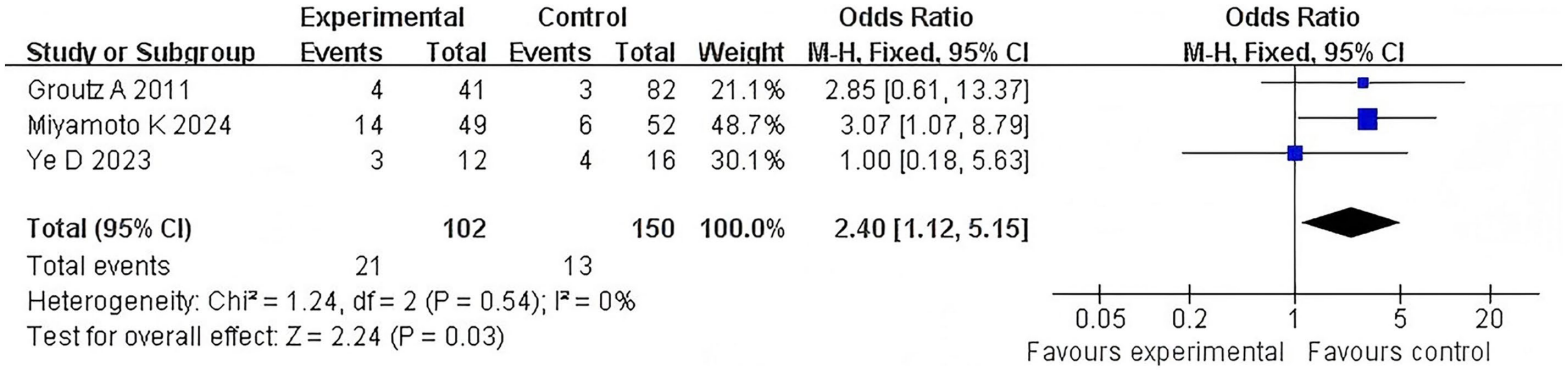
Urinary retention comparison forest map.

Three articles examined the correlation between the duration of the second stage of labor in primiparous women and recent postpartum urinary retention. The *Q*-test and *I*^2^-test showed *p* = 0.54 (chi-squared = 1.24, df = 4) and *I*^2^ = 0%, indicating no heterogeneity among the studies. The results showed no significant difference between the two groups (*Z* = 2.24, *p* = 0.03). Moreover, urinary retention was more severe in the extension group than in the control group (OR = 2.40, 95% CI (1.12–5.15), *p* = 0.03). In Figure 8, the forest plot shows the association between prolonged second stage of labor and urinary retention. An OR greater than 1 indicates an increased risk of urinary retention in women with a prolonged second stage of labor. The diamond in the plot represents the pooled odds ratio. See Figure 8 for details.

## 4 Discussion

The structure of the pelvic floor is complex, which supports the pelvic organs. In the second stage of labor, with an increase of time, the pressure borne by the pelvic floor muscles and fascia increases, which will lead to a series of changes, such as damaged muscle fibers, reduced fascia elasticity, and nerve conduction disorders, further triggering pelvic floor dysfunction. Such injuries can present as pain, urinary incontinence, urinary retention, and similar symptoms in the short term after delivery and increase the risks of pelvic organ prolapse, fecal incontinence, and sexual dysfunction in the long term. Therefore, studying the correlation between the duration of the second stage of labor and pelvic floor function impairment in primiparous women is of great significance for guiding clinical practice and formulating a postpartum rehabilitation program.

The meta-analysis results revealed that the UI in the extension group was more severe [OR = 1.95, 95% CI (1.30–2.92), *p* = 0.001]. Our findings are consistent with a previous meta-analysis by Stafne et al. (12), which also reported a correlation between prolonged second stage of labor and urinary incontinence. However, our study only included randomized controlled trials (RCTs), and the evidence was stronger. During the second stage of labor, the pelvic floor muscles and urethral sphincter were able to maintain the normal closure function of the urethra and ensure urine control. However, when labor prolonged, the pelvic floor muscles and the urethral sphincter were subjected to pressure generated by the fetus through the birth canal for a long time, and this excessive pressure led to structural and functional damage to the urethral sphincter. From the perspective of muscle physiology, long-term pressure makes the pelvic floor muscles tense continuously, causing muscle fatigue and muscle strength decline, which, in turn, affects their ability to support and control the urethra (12). For primiparous parturients with prolonged second labor, postpartum prevention and treatment measures for urinary incontinence should be actively carried out, such as guiding the parturients to conduct standardized pelvic floor muscle training, and improving the urethral sphincter function by enhancing the pelvic floor muscle strength, thereby reducing the symptoms of urinary incontinence and improving the quality of life of the parturients. The condition of pelvic floor pain in the extension group was stronger [OR = 1.24, 95% CI (1.03–1.50), *p* = 0.03] because, in the extended second stage of labor, the pelvic floor muscles, fascia, and nerves were constantly in tension and compression. Long-term tension of the pelvic floor muscles led to the accumulation of intramuscular metabolites, especially lactic acid, which caused local blood circulation disorders, leading to muscle ischemia and hypoxia. These changes stimulated nerve endings and caused pain (13). In addition, under long-term overstretching, fascia is prone to slight tearing and inflammatory reactions, further aggravating pain sensation. When the nerves are compressed and pulled, their conduction function is damaged, which enhances and sustains pain signals. From the perspective of clinical treatment, medical staff should pay close attention to postpartum pelvic floor pain. Various physical therapies can be used, such as thermal therapy to promote pelvic floor blood circulation, relieve muscle tension, and reduce pain, as well as electrical stimulation technology to repair damaged nerve function and improve nerve conduction. At the same time, psychological intervention is indispensable to help pregnant women relieve anxiety caused by pain and improve their tolerance to pain. Regarding urinary retention, the condition was more severe in the EA group [OR = 2.40, 95% CI (1.12–5.15), and *p* = 0.03]. This is primarily due to prolonged compression of the bladder and urethra during the prolonged second stage of labor. During the normal process of delivery, the bladder is compressed to a certain extent. However, when the stage of labor is prolonged, the compression time also becomes extended, which will make the bladder mucosa ischemic and hypoxic, impair the bladder’s sensory function and contractility, resulting in the bladder being not effectively emptied (14). At the same time, the tissues around the urethra may be damaged due to long-term compression, causing urethral obstruction and increasing urination resistance. In clinical practice, for parturients experiencing prolonged second labor, medical staff should pay close attention to their urination. Once signs of urinary retention are found, effective treatment measures should be taken in time, such as inducing urination or intermittent catheterization, to avoid complications such as urinary system infection caused by urinary retention and to protect the normal function of the bladder and urethra.

Although there was no significant difference in POP [OR = 1.25, 95% CI (0.99–1.59), *p* = 0.06] and FI [OR = 1.28, 95% CI (0.98–1.68), *p* = 0.07] values between the extension group and the general group, the OR values of the two showed a trend. The results differ from those of Bergendahl et al. (15) who concluded that the prolongation of the second stage of labor is not associated with fecal incontinence. Although this study has not reached a significant conclusion, it is observed that the risk of fecal incontinence is increasing. This may be due to the larger sample size of this study, which allowed for detecting weak associations, as well as stricter inclusion criteria, which reduces the influence of confounding factors. Compared with Huber et al. (16), who focused on changes in pelvic floor function within 1 year after delivery, this study focused on the recent pelvic floor function damage occurring within 6 weeks after delivery, with different periods and different results. Huber et al. (16) found that, over time after delivery, some pelvic floor dysfunction tends to alleviate, but this study mainly shows the influence of prolonged second stage of labor on pelvic floor function in the short term after delivery. These differences provide a direction for further research to further clarify the relationship between them. Pelvic floor support structures (including pelvic floor muscles and fascia, ligaments) are subjected to excessive stress during the second stage of labor extension, which may potentially damage these support structures. Although no significant differences in POP and FI have been observed in recently in this study, this damage may gradually manifest over time, increasing the risk of pelvic organ prolapse and fecal incontinence. Therefore, clinicians need to be vigilant against these potential problems during the follow-up of primiparous parturients after delivery. For parturients with related symptoms, detailed evaluation and further examination should be conducted to identify and treat possible pelvic floor dysfunction at an early stage. Postpartum sexual dysfunction was not significantly worse in the extension group compared with the control group [OR = 1.67, 95% CI (0.81–3.42), *p* = 0.16]. This finding indicates that postpartum sexual dysfunction is a complex problem affected by a combination of multiple factors. Although the pelvic floor tissues may suffer some damage during the prolongation of the second stage of labor, this is not the only factor affecting sexual function. Our findings suggest that clinicians should closely monitor primiparous women with a prolonged second stage of labor for early signs of pelvic floor dysfunction, particularly urinary incontinence and pelvic pain. Prophylactic pelvic floor muscle training should be considered for this high-risk group. Furthermore, these findings should be discussed with women during antenatal counseling to inform them about the potential risks associated with prolonged labor.

Although 13 articles were included in this study and there were 2,862 researchers in total, the sample size may still be insufficient for some more detailed analyses. For example, in further exploring the relationship between the difference in pelvic floor function impairment and the duration of the second stage of labor among different subgroups (such as primiparous women of different ages and with different body mass indices), a larger sample size may be needed to improve statistical efficiency to obtain more accurate and reliable results. In addition, the main concern of this study was recent postpartum (within 6 weeks) pelvic floor dysfunction. However, pelvic floor function may undergo dynamic changes over a longer period of time after delivery, and the manifestations and extent of pelvic floor impairment may vary with the further recovery of the maternal body, the adjustment of hormone levels, and lifestyle changes. The lack of long-term follow-up data limits our overall understanding of the long-term relationship between labor duration and pelvic floor function impairment and may result in inadequate in-depth and accurate interpretation of some findings. Our meta-analysis is limited by the heterogeneity in how the “prolonged second stage of labor” is defined across the included studies, which may have introduced variability into the results. Additionally, the inclusion of studies with a moderate risk of bias could have affected the certainty of the effect estimates. The majority of studies focused on short-term outcomes, and the long-term consequences of a prolonged second stage on pelvic floor health remain unclear. The small number of included studies for certain outcomes (e.g., urinary retention, *n* = 3) precluded formal assessment of publication bias via funnel plots or Egger’s test, potentially underpowering our conclusions. Although sensitivity analyses were performed for heterogeneous outcomes, residual confounding from unmeasured variables (e.g., ethnicity and postpartum rehabilitation protocols) may persist. Future research should focus on large, prospective cohort studies that use standardized definitions of prolonged second stage of labor and employ validated tools to assess pelvic floor function at multiple time points postpartum. These studies should investigate the influence of specific interventions during the second stage (e.g., different pushing techniques and the use of vacuum extraction) on the risk of pelvic floor injury. Furthermore, research should explore the underlying mechanisms linking prolonged labor to pelvic floor damage, such as the role of excessive stretching and ischemia.

In summary, the results of this meta-analysis showed that the duration of the second stage of labor was significantly correlated with urinary incontinence, pelvic floor pain, and urinary retention, with a potential impact on pelvic organ prolapse and fecal incontinence, but not with postpartum sexual dysfunction.

## Data Availability

The raw data supporting the conclusions of this article will be made available by the authors, without undue reservation.

## References

[1] Cohen WR Friedman EA. The second stage of labor. Am J Obstet Gynecol. (2024) 230:S865–75. doi: 10.1016/j.ajog.2022.06.014, PMID: 38462260

[2] Schütze S Heinloth M Uhde M Schütze J Hüner B Janni W . The effect of pelvic floor muscle training on pelvic floor function and sexuality postpartum. A randomized study including 300 primiparous. Arch Gynecol Obstet. (2022) 306:785–93. doi: 10.1007/s00404-022-06542-z, PMID: 35377043 PMC8977567

[3] Del Forno S Cocchi L Arena A Pellizzone V Lenzi J Raffone A . Effects of pelvic floor muscle physiotherapy on urinary, bowel, and sexual functions in women with deep infiltrating endometriosis: a randomized controlled trial. Medicina. (2023) 60:67. doi: 10.3390/medicina60010067, PMID: 38256327 PMC10818504

[4] Collins S Lewicky-Gaupp C. Pelvic organ prolapse. Gastroenterol Clin N Am. (2022) 51:177–93. doi: 10.1016/j.gtc.2021.10.011, PMID: 35135661

[5] Tunn R Baessler K Knüpfer S Hampel C. Urinary incontinence and pelvic organ prolapse in women. Dtsch Arztebl Int. (2023) 120:71–80. doi: 10.3238/arztebl.m2022.0406, PMID: 36647585 PMC10080228

[6] Menees SB Lembo A Charabaty A. Fecal incontinence and diarrhea during pregnancy. Am J Gastroenterol. (2022) 117:26–32. doi: 10.14309/ajg.0000000000001964, PMID: 36194030

[7] Cosgriff L Ramanathan A Iglesia CB. Pelvic floor disorders and sexual function: a review. Obstet Gynecol Clin N Am. (2024) 51:241–57. doi: 10.1016/j.ogc.2024.02.001, PMID: 38777481

[8] Peinado-Molina RA Hernández-Martínez A Martínez-Vázquez S Rodríguez-Almagro J Martínez-Galiano JM. Pelvic floor dysfunction: prevalence and associated factors. BMC Public Health. (2023) 23:2005. doi: 10.1186/s12889-023-16901-3, PMID: 37838661 PMC10576367

[9] Nutaitis AC Meckes NA Madsen AM Toal CT Menhaji K Carter-Brooks CM . Postpartum urinary retention: an expert review. Am J Obstet Gynecol. (2023) 228:14–21. doi: 10.1016/j.ajog.2022.07.060, PMID: 35932877

[10] Wright D Wright A Tan MY Nicolaides KH. When to give aspirin to prevent preeclampsia: application of Bayesian decision theory. Am J Obstet Gynecol. (2022) 226:S1120–5. doi: 10.1016/j.ajog.2021.10.038, PMID: 35177216

[11] MacCraith E Cunnane EM Joyce M Forde JC O’Brien FJ Davis NF. Comparison of synthetic mesh erosion and chronic pain rates after surgery for pelvic organ prolapse and stress urinary incontinence: a systematic review. Int Urogynecol J. (2021) 32:573–80. doi: 10.1007/s00192-020-04612-x, PMID: 33237357

[12] Stafne SN Dalbye R Kristiansen OM Hjelle YE Salvesen KÅ Mørkved S . Antenatal pelvic floor muscle training and urinary incontinence: a randomized controlled 7-year follow-up study. Int Urogynecol J. (2022) 33:1557–65. doi: 10.1007/s00192-021-05028-x, PMID: 34936023 PMC9206614

[13] Worman RS Stafford RE Cowley D Prudencio CB Hodges PW. Evidence for increased tone or overactivity of pelvic floor muscles in pelvic health conditions: a systematic review. Am J Obstet Gynecol. (2023) 228:657–674.e91. doi: 10.1016/j.ajog.2022.10.027, PMID: 37272325

[14] Rodaki E Diamanti A Sarantaki A Lykeridou A. The effects of perineal tears during childbirth on women’s sex life. Maedica. (2022) 17:297–305. doi: 10.26574/maedica.2022.17.2.297, PMID: 36032614 PMC9375874

[15] Bergendahl S Sandström A Spasojevic A Brismar Wendel S. Anal incontinence after a prolonged second stage of labor in primiparous women. Sci Rep. (2022) 12:7315. doi: 10.1038/s41598-022-11346-x, PMID: 35513490 PMC9072350

[16] Huber M Malers E Tunón K. Pelvic floor dysfunction one year after first childbirth in relation to perineal tear severity. Sci Rep. (2021) 11:12560. doi: 10.1038/s41598-021-91799-8, PMID: 34131194 PMC8206367

[17] McKenzie CM Woolfolk CL Rieger MM White AB Tuuli MG Srinivas SK . Impact of the duration of the second stage of labor on postpartum pelvic floor symptoms. Urogynecol. (2024) 30:381–7. doi: 10.1097/SPV.0000000000001477, PMID: 38484257 PMC10947063

[18] Tuuli MG Gregory WT Arya LA Lowder JL Woolfolk C Caughey AB . Effect of second-stage pushing timing on postpartum pelvic floor morbidity: a randomized controlled trial. Obstet Gynecol. (2023) 141:245–52. doi: 10.1097/AOG.0000000000005031, PMID: 36603202

[19] Gimovsky AC Phillips JM Amero M Levine J Berghella V. Prolonged second stage effect on pelvic floor dysfunction: a follow up survey to a randomized controlled trial. J Matern Fetal Neonatal Med. (2022) 35:5520–5. doi: 10.1080/14767058.2021.1887122, PMID: 33586572

[20] Mevorach Zussman N Gonen N Kovo M Miremberg H Bar J Condrea A . Protracted postpartum urinary retention-a long-term problem or a transient condition. Int Urogynecol J. (2020) 31:513–9. doi: 10.1007/s00192-019-03903-2, PMID: 30783707

[21] Van Kessel K Reed S Newton K Meier A Lentz G. The second stage of labor and stress urinary incontinence. Am J Obstet Gynecol. (2001) 184:1571–5. doi: 10.1067/mob.2001.114856 11408883

[22] Koyucu RG Demirci N. Effects of pushing techniques during the second stage of labor: a randomized controlled trial. Taiwan J Obstet Gynecol. (2017) 56:606–12. doi: 10.1016/j.tjog.2017.02.005, PMID: 29037544

[23] Badiou W Bousquet PJ Prat-Pradal D Monroziès X Mares P de Tayrac R. Short vs long second stage of labour: is there a difference in terms of postpartum anal incontinence. Eur J Obstet Gynecol Reprod Biol. (2010) 152:168–71. doi: 10.1016/j.ejogrb.2010.06.004, PMID: 20650561

[24] O’Leary BD Keane DP. Effect of the length of the second stage of labor on pelvic floor dysfunction. Am J Obstet Gynecol. (2023) 5:100795. doi: 10.1016/j.ajogmf.2022.100795, PMID: 36334722

[25] Jin X Wu S Huang J Tong X Li H Chu L. Effect of prolonged second stage of labor on pelvic floor function: a prospective cohort study. Int Urogynecol J. (2022) 33:1633–8. doi: 10.1007/s00192-022-05136-2, PMID: 35267059

[26] Pardo E Rotem R Glinter H Erenberg M Yahav L Yohay Z . Recovery from pelvic floor dysfunction symptoms in the postpartum is associated with the duration of the second stage of labor. Arch Gynecol Obstet. (2019) 300:127–33. doi: 10.1007/s00404-019-05173-1, PMID: 31053946

[27] Groutz A Levin I Gold R Pauzner D Lessing JB Gordon D. Protracted postpartum urinary retention: the importance of early diagnosis and timely intervention. Neurourol Urodyn. (2011) 30:83–6. doi: 10.1002/nau.20926, PMID: 20860036

[28] Miyamoto K Komatsu H Nagata H Nagira K Motomura E Shimizu N . Prolonged second stage of labor in delivery using epidural analgesia is a risk factor for postpartum urinary retention. J Obstet Gynaecol Res. (2024) 50:424–9. doi: 10.1111/jog.15867, PMID: 38124232

[29] Ye D Yao LQ. Prolonged second stage of labor is associated with persistent urinary retention after forceps delivery: an observational study. Medicine. (2023) 102:e35169. doi: 10.1097/MD.0000000000035169, PMID: 37746990 PMC10519570

